# The human phylome

**DOI:** 10.1186/gb-2007-8-6-r109

**Published:** 2007-06-13

**Authors:** Jaime Huerta-Cepas, Hernán Dopazo, Joaquín Dopazo, Toni Gabaldón

**Affiliations:** 1 Bioinformatics Department, Centro de Investigación Príncipe Felipe, Autopista del Saler, 46013 Valencia, Spain.

## Abstract

**Background:**

Phylogenomics analyses serve to establish evolutionary relationships among organisms and their genes. A phylome, the complete collection of all gene phylogenies in a genome, constitutes a valuable source of information, but its use in large genomes still constitutes a technical challenge. The use of phylomes also requires the development of new methods that help us to interpret them.

**Results:**

We reconstruct here the human phylome, which includes the evolutionary relationships of all human proteins and their homologs among 39 fully sequenced eukaryotes. Phylogenetic techniques used include alignment trimming, branch length optimization, evolutionary model testing and maximum likelihood and Bayesian methods. Although differences with alternative topologies are minor, most of the trees support the Coelomata and Unikont hypotheses as well as the grouping of primates with laurasatheria to the exclusion of rodents. We assess the extent of gene duplication events and their relationship with the functional roles of the protein families involved. We find support for at least one, and probably two, rounds of whole genome duplications before vertebrate radiation. Using a novel algorithm that is independent from a species phylogeny, we derive orthology and paralogy relationships of human proteins among eukaryotic genomes.

**Conclusion:**

Topological variations among phylogenies for different genes are to be expected, highlighting the danger of gene-sampling effects in phylogenomic analyses. Several links can be established between the functions of gene families duplicated at certain phylogenetic splits and major evolutionary transitions in those lineages. The pipeline implemented here can be easily adapted for use in other organisms.

## Abstract 2

The human phylome, which includes evolutionary relationships of all human proteins and their homologs among thirty-nine fully sequenced eukaryotes, is reconstructed.

## Background

The complete sequencing of the human genome represented a major breakthrough for the genome era [1,2]. Since then, a number of genome wide experimental and computational analyses have been performed that capture different aspects of the biology of the human cell. These analyses include, among many others, those of the so-called transcriptome [3], proteome [4], interactome [5] and metabolome [6]. The availability of such large datasets have added new dimensions to the study of the human organism; not only are they useful in elucidating the function of otherwise uncharacterized proteins, but they also provide information on the system-level properties of the cell [7]. The reconstruction of the evolutionary histories of all genes encoded in a genome, the so-called phylome [8], constitutes another source of genome-wide information. Analyses of complete phylomes, however, have traditionally been prevented by their large demands on time and computer power. Only recently have faster computers and algorithms paved the way for the application of phylogenetics to whole genomes. Such analyses have proven to be a very useful tool for the detection of specific evolutionary scenarios [9] and for the functional characterization of genes and biological systems [10,11]. Other large-scale phylogenetic analyses have focused on the establishment of orthology relationships among genes in model species. Most remarkably, the Ensembl database now includes phylogenetic trees [12], and the TreeFam [13] and HOVERGEN [14] databases provide automatically derived and curated phylogenies of animal gene families. Other such databases focus on specific aspects of the evolution of gene families, such as the detection of adaptive events [15]. These databases follow a family-based approach, since they first group the genes into families and subsequently build a single phylogeny for each family.

**Figure 1:**
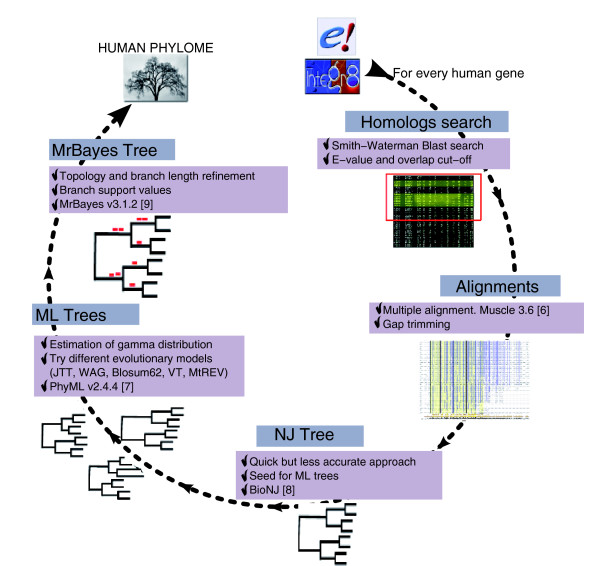
Schematic representation of the phylogenetic pipeline used to reconstruct the human phylome. Each protein sequence encoded in the human genome is compared against a database of proteins from 39 fully sequenced eukaryotic genomes (Table 1) to select putative homologous proteins. Groups of homologous sequences are aligned and subsequently trimmed to remove gap-rich regions. The refined alignment is used to build a NJ tree, which is then used as a seed tree to perform a ML likelihood analysis as implemented in PhyML, using four different evolutionary models (five in the case of mitochondrially encoded proteins). The ML tree with the maximum likelihood is further refined with a Bayesian analysis using MrBayes. Finally, different algorithms are used to search for specific topologies in the phylome or to define orthology and paralogy relationships.

Using a different, gene-based approach that aims at maximizing both the coverage over the human genome and the taxon-sampling among fully sequenced eukaryotic genomes, we have developed a fully automated pipeline (Figure 1) to reconstruct the phylogenies of every protein encoded in the human genome and its homologs in 39 eukaryotic species. Such a pipeline aims at resembling, as much as possible, the manual procedure used by phylogeneticists while remaining a fully automated process. In the search for a compromise between time and reliability, we always tried to adjust the balance towards the latter, thus assuring high quality in the resulting phylogenies. In contrast to the abovementioned TreeFam and Ensembl phylogenetic pipelines, our approach includes evolutionary model testing using maximum likelihood (ML), model parameter estimation and alignment trimming steps. Moreover, besides using neighbor joining (NJ) and ML approaches for phylogenetic reconstruction, our pipeline also implements a Bayesian phylogenetic reconstruction approach to provide posterior probabilities of every partition in the tree. As a result, building the human phylome presented here took two months on a total of 140 64-bit processors, which is roughly equivalent to 23 years in a single processor. To our knowledge, this represents the most sophisticated phylome reconstruction pipeline and the largest computing time investment for a single phylome reported to date.

The availability of such a comprehensive collection of evolutionary histories of protein-coding human genes constitutes a valuable source of information that allows us to test several evolutionary hypotheses. For this purpose, we investigated the consistency of the individual phylogenies within the phylome with alternative evolutionary scenarios, namely those involving the relative positions of rodents and primates, amoebozoans and opisthokonts and, finally, insects, nematodes and chordates. We also scanned the human phylome for cases of putative horizontally transferred genes and found that such topologies are never highly supported, indicating that they are rather the result of phylogenetic artifacts. Moreover, we provide estimates for the number of gene duplications that have occurred at different evolutionary stages in the eukaryotic lineages leading to hominids and found several over-represented functional classes in the different duplication events. Finally, we explored an alternative, fully automated algorithm to infer orthology relationships from phylogenetic trees that does not require a fully resolved species phylogeny and, therefore, is less sensitive to topological variations. The choice for this novel methodology for orthology prediction is based on the fact that alternative tree reconciliation methods have difficulties in accounting for inherent phylogenetic noise, divergences in evolutionary histories for different genes and the low resolution level of available species trees. As will be shown below, the high degree of topological variation found in the human phylome for all scenarios considered also supports the choice of alternatives to classic tree reconciliation methods. All in all, the results presented here constitute a preliminary but broad overview of the evolutionary history of the human genome, which is not taken as an average or represented by a limited number of genes, but instead is regarded as a complex mosaic of thousands of individual phylogenies.

## Results and discussion

### Phylome scope and phylogenetic pipeline

The human phylome presented here is derived from the proteins encoded by 39 publicly available eukaryotic genomes (Table 1). This set is particularly rich in metazoan species (19 species, 50%), including 14 chordates, 3 arthropods and 2 nematodes. The second largest group is that of fungi, comprising 11 species and thus making a total of 30 opisthokons. The remaining group includes eight species from diverse phyla, among which are one amoebozoan (*Dictyostellum discoideum*), two plants (*Arabiopsis thaliana *and *Chlamydomonas reinhardti*), two apicomplexans (*Plasmodium falciparum *and *Plasmodium briggsae*), and three excavates (the diplomonad *Guillardia theta *and the kinetoplastids *Leishmania major *and *Paramecium tetraurelia*). This distribution of species makes our set especially suitable for addressing the evolution of protein families among the opisthokonts. It covers, therefore, a period that is rich in important evolutionary innovations, from the origin of apoptotic pathways [16] to the emergence of complex communication patterns [17].

**Table 1:** Species included in the present phylome and their genomic coverage

Group	Code	Species name	Source	Proteins included (%)	Trees (%)
Primates	Hsa	*Homo sapiens*	Ensembl	21,726 (99.1%)	21,588 (100.0%)
	Ptr	*Pan troglodytes*	Ensembl	17,113 (79.3%)	19,577 (90.7%)
	Mmu	*Macaca mulatta*	Ensembl	19,285 (89.2%)	19,765 (91.6%)
					
Placental mammals	Mms	*Mus musculus*	Ensembl	19,934 (78.9%)	18,825 (87.2%)
	Rno	*Rattus norvegicus*	Ensembl	18,675 (85.7%)	18,585 (86.1%)
	Cfa	*Canis familiaris*	Ensembl	16,657 (91.8%)	18,834 (87.2%)
	Bta	*Bos taurus*	Ensembl	18,457 (79.9%)	18,736 (86.8%)
					
Mammals	Mdo	*Monodelphis domestica*	Ensembl	17,004 (80.7%)	18,013 (83.4%)
					
Vertebrates	Gga	*Gallus gallus*	Ensembl	12,325 (66.5%)	15,758 (73.0%)
	Xtr	*Xenopus tropicalis*	Ensembl	14,721 (60.6%)	15,787 (73.1%)
	Tni	*Tetraodon nigroviridis*	Ensembl	14,896 (53.4%)	14,585 (67.6%)
	Fru	*Fugu rubripes*	Ensembl	15,834 (72.3%)	15,155 (70.2%)
	Dre	*Danio rerio*	Ensembl	16,042 (74.9%)	14,808 (68.6%)
					
Chordates	Cin	*Ciona intestinalis*	Ensembl	5,588 (50.9%)	9,421 (43.6%)
					
Metazoa	Aga	*Anopheles gambiae*	Ensembl	6,131 (43.0%)	9,310 (43.1%)
	Dme	*Drosophila melanogaster*	Ensembl	6,812 (49.6%)	9,771 (45.3%)
	Ame	*Apis mellifera*	Ensembl	4,484 (33.4%)	8,616 (39.9%)
	Cel	*Caenorhabditis elegans*	Ensembl	5,826 (29.8%)	8,190 (37.9%)
	Cbr	*Caenorhabditis briggsae*	Integr8	5,171 (39.2%)	7,899 (36.6%)
					
Opisthokonts	Ago	*Ashbya gossypii*	Integr8	2,020 (42.8%)	3,603 (16.7%)
	Cal	*Candida albicans*	Other	2,733 (33.8%)	3,899 (18.1%)
	Cgl	*Candida glabrata*	Integr8	2,129 (41.1%)	3,627 (16.8%)
	Cne	*Cryptococcus neoformans*	Integr8	2,532 (38.5%)	4,102 (19.0%)
	Dha	*Debaromyces hansenii*	Integr8	2,302 (36.5%)	3,885 (18.0%)
	Ecu	*Encephalitozoon cuniculi*	Integr8	626 (32.8%)	1,203 (5.6%)
	Gze	*Giberella zeae*	Integr8	3,076 (26.4%)	4,412 (20.4%)
	Kla	*Kluyveromyces lactis*	Integr8	2,077 (39.1%)	3,715 (17.2%)
	Ncr	*Neurospora crassa*	Other	2,521 (23.7%)	4,221 (19.6%)
	Sce	*Saccharomyces cerevisiae*	Ensembl	2,317 (35.1%)	3,769 (17.5%)
	Spb	*Schizosaccharomyces pombe*	Integr8	2,421 (48.8%)	4,102 (19.0%)
	Yli	*Yarrowia lipolytica*	Integr8	2,487 (38.1%)	4,152 (19.2%)
					
Amoebozoa	Ddi	*Dictyostelium discoideum*	Integr8	3,843 (29.4%)	5,165 (23.9%)
					
Plants	Ath	*Arabidopsis thaliana*	Integr8	9,450 (26.6%)	5,390 (25.0%)
	Cre	*Chlamydomonas reinhardtii*	Other	2,303 (11.7%)	3,504 (16.2%)
					
Diplomonad	Gth	*Gillardia theta*	Integr8	161 (35.7%)	458 (2.1%)
					
Apicomplexa	Pfa	*Plasmodium falciparum*	Integr8	1,330 (25.3%)	2,507 (11.6%)
	Pyo	*Plasmodium yoelii*	Integr8	1,188 (15.3%)	2,272 (10.5%)
					
Kinetoplastida	Lma	*Leishmania major*	Integr8	2,082 (26.0%)	3,130 (14.5%)
	Pte	*Paramecium tetraurelia*	Integr8	140 (30.2%)	345 (1.6%)

For each species: the 'Proteins included' column indicates the number of proteins present in trees of the human phylome and the percentage they represent; and the 'Trees' column indicates the number of trees in the phylome with proteins from that species (and the percentage from the phylome it represents). 'Source' indicates the database from which the protein data for that species were retrieved.

To derive a phylome from the abovementioned proteome database we applied a phylogenetic pipeline to each human protein. This fully automated pipeline (described in more detail in the Materials and methods section) emulates the manual workflow used by phylogeneticists: from sequence, through alignment, to phylogenetic reconstruction. It starts with a sequence search against the proteome database to retrieve groups of significantly similar proteins that are then aligned. Alignments are automatically trimmed to remove gap-rich regions. The subsequent phylogenetic reconstruction combines NJ, ML and Bayesian methods. Firstly, a NJ tree is constructed with BioNJ [18], and secondly, this NJ tree is used as a seed in a ML analysis using PhyML [19]. In the ML analysis, up to five different evolutionary models were tested for each tree (see below) using a discrete gamma-distribution model with four rate categories plus invariant positions. Both the gamma shape parameter and the fraction of invariant positions were estimated from the data. Finally, the ML tree rendered by the model best fitting the data, as determined by the Akaike Information Criterion (AIC) [20], was further refined with a Bayesian approach as implemented in MrBayes [21]. After the Bayesian analysis, a consensus tree was produced by using the 'halfcompat' option of MrBayes, which produces a topology in which all partitions are compatible with at least 50% of the trees produced by the Monte Carlo Markov Chain analysis (see Materials and methods). Unless stated otherwise, this tree was used in all subsequent analyses. The resulting 21,588 alignments and 129,510 trees from the different phylogenetic approaches are available as supplementary material accompanying this article [22].

### Evolutionary model selection

Both ML and Bayesian analyses are model-based approaches that can provide divergent results when different evolutionary models are assumed. Several authors have shown that the use of an appropriate model is crucial for the reconstruction of correct phylogenies and that the origin of the sequences involved (that is, the range of organisms involved) is not always a good predictor of the most appropriate model [23,24]. Applying a wrong evolutionary model to a given dataset might even lead to the reconstruction of wrong phylogenies with a high support [25]. To avoid such pitfalls, we tested using the ML approach several models that are complementary in their scope, namely: JTT [26], a general model for globular, nuclear-encoded proteins; BLOSUM62 [27], inferred from protein blocks of 62% sequence identity; WAG, derived from a database of globular proteins with a broad range of evolutionary distances [28]; and VT, based on amino acid replacement rates suited for distantly related sequences [29]. Additionally, phylogenies of the proteins encoded in the mitochondrial genome were also reconstructed using mtREV, a model that has been specifically designed for this kind of data [30]. In all cases, a discrete gamma-distribution model with four rate categories plus invariant positions was used. The gamma parameter and the fraction of invariant positions were estimated from the data.

Among the models tested, JTT was chosen as the best fitting model in a majority of the trees (14,683, 68.0%), followed by WAG (6,388, 29.6%), Blosum62 (461, 2.1%) and VT (26, 0.1%). MtREV was chosen as the best model in ten out of the thirteen mitochondrial-encoded human proteins. Surprisingly, the phylogenies of subunit 6 of NADH dehydrogenase and subunits 1 and 2 of cytochrome oxidase were best fitted by JTT, Blosum62 and WAG models, respectively.

To assess whether a tree produced by the NJ approach has sufficient predictive value for the model selection step, we compared the model chosen by the full ML approach (that is, reconstructing a ML phylogeny for every model) to the model selected when the likelihood of the seed NJ tree was assessed under different models, allowing for branch-length optimization. In 86.7% of the cases the model chosen by both methods was the same. This confirms and extends earlier results [23] and, more importantly, suggests that the pipeline can be simplified by basing the model selection on the tree produced by BioNJ.

### The tree of eukaryotes and the topological diversity within the human phylome

Recent advances in resolving the tree of eukaryotes are converging into a model that comprises a few large super-groups [31]. Despite the general agreement on the classification of these major groups, several relationships, both among and within the different groups, remain controversial. In recent years, a number of large-scale approaches have been developed that combine the information obtained from several genes to resolve evolutionary relationships. Among these, the construction of super-trees and trees based on concatenated alignments are among the most widely used [32]. These trees are useful in that they constitute a straightforward way of visualizing the combined phylogenetic signal of genes that are widespread in the species considered. However, it has been claimed that these trees are representative of only a small fraction of the genes encoded in a given genome, and that gene-sampling effects might lead to biased results supporting a specific species phylogeny [33,34].

**Figure 2:**
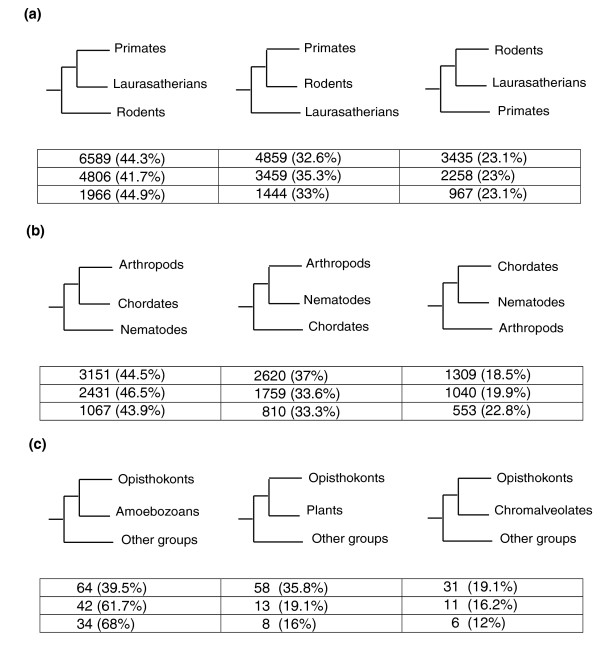
The alternative phylogenetic relationships among the taxa involved in the three evolutionary hypotheses considered. **(a) **Placental mammals: primates, laurasatheria and rodents. **(b) **Ecdysozoa versus Coelomata hypothesis: relationships among arthropods, chordates and nematodes. And **(c) **the Unikont hypothesis: relationship among opisthokonts, amoebozoans and other eukaryotic groups. The numbers indicate the number of trees supporting each topology. For each alternative topology numbers on the top row refer to the total number of trees with a given topology, and what percentage of the total it represents; numbers in the middle row refer to those trees for which the posterior probabilities of the two partitions shown in the figure are 0.9 or higher. Numbers in the bottom row refer to the number and percentage of gene families supporting each topology.

A phylome represents a broader, yet more complex to interpret, reconstruction of the evolution of an organism, since it comprises the phylogenies of all its genes. Most notably, the availability of a phylome opens the possibility for studying the relationships among species in a different way: that of quantifying the fraction of individual phylogenies whose topologies are consistent with a given hypothesis. Here we explored this methodology by specifically contrasting a number of evolutionary relationships that are controversial to some extent. We chose three different scenarios for which there is some level of controversy in the literature and that involve three different depths of the eukaryotic tree (Figure 2). Namely, the relative positions of nematodes, chordates and arthropods, the relationships among rodents, primates and laurasatherians, and, lastly, the grouping of opisthokonts with amoebozoans. To scan for phylogenies compatible with the different hypotheses, we adapted a previously described algorithm [9] (see also Materials and methods).

### Ecdysozoa versus coelomata hypotheses

Perhaps one of the most debated issues regarding the tree of eukaryotes is the relative position of arthropods, nematodes and chordates. Traditionally, comparative anatomy placed arthropods and chordates in the coelomata clade, which contained animals with a true body cavity, while pseudocoelomates such as nematodes occupied a more basal position. However, phylogenetic analyses of 18S and 28S rRNAs supported an alternative view that grouped nematodes and arthropods, dubbed ecdysozoa, to the exclusion of chordates [35]. Since then, numerous multi-gene phylogenetic studies that support either of the hypotheses have been published (see, among others, [36-39]).

Our results (Figure 2) show a preponderance of genes whose phylogeny is consistent with the Coelomata hypothesis. Of the 7,080 phylogenies in the human phylome with representatives from the three groups, 3,151 (44.5%) support the Coelomata hypothesis, placing nematodes at a basal position, compared to 2,620 (37%) and 1,309 (18.5%) that group nematodes with arthropods (Ecdysozoa hypothesis) or with chordates, respectively. The relative fraction of trees supporting each topology is similar if we consider only the 5,230 trees with the highest topology support (posterior probabilities higher than 0.9 in the nodes grouping the considered taxa (Figure 2). Since the algorithm treats each gene individually, a certain level of redundancy exists because protein families with many members in the human genome contribute more trees to the phylome. These would affect the topological analysis if there are great differences in the distribution of family sizes supporting each topology. To correct for this redundancy we grouped the individual gene-trees into families if their seed sequences appeared together in a tree. Then each family was considered to support a single topology. If more than a single topology was supported, the one supported by a majority of members was chosen. As shown in Figure 2 (bottom row), the percentage of families supporting each topology is similar to the results obtained when genes are treated individually.

The finding that all three possible topologies, including the one widely considered as wrong in the literature, are supported by a significant number of trees illustrates the inherent difficulty of resolving the species phylogeny from gene phylogenies. We have found similar topological diversity in the three scenarios considered (see below) and also, to smaller degrees, in apparently undisputed evolutionary relationships (results not shown). Similar results showing variability in the relative positions of arthropods, nematodes and chordates have also been found in topological analyses of the phylogenies of 507 eukaryotic orthologous groups [38] and of 100 protein families [40]. These deviances from the species phylogeny might be the result of different processes, including convergent evolution or varying evolutionary rates. In the case of the Ecdysozoa and Coelomata hypotheses, the accelerated rate of evolution in the nematode sequences has been proposed as the main cause preventing the acceptance of the Ecdysozoa hypothesis. For instance, some studies have shown that when fast evolving genes are removed from the dataset, the ecdysozoa group is accepted with high confidence [36,39]. Therefore, the relative abundance of the different topologies should be considered with caution, since differences in evolutionary rates, if they are widespread, could result in a majority of the gene trees supporting a wrong species phylogeny.

### Relationships among placental mammals

The phylogenetic relationship among placental mammals has attracted great interest in recent years [41]. A still open question is the relative grouping and branching order of the groups rodentia, primates, lagomorpha, artyodactyla and carnivora. Four of these groups are represented in the present phylome, namely primates (human, chimpanzee and macaque), artyodactyla (cow), carnivora (dog) and rodents (rat and mouse). While the monophily of artyodactyla and carnivores, both belonging to laurasatheria, is largely undisputed, the crucial question is whether rodents have a basal position relative to the other groups or whether they join primates on a common node. Analyses of concatenated alignments from nuclear genes are consistent with the rodents being a basal group and primates being monophyletic with laurasatheria [42,43]. However, phylogenies based on mitochondrial genes as well as the common presence of several mutational events and the insertion of MLTA0 elements support the clustering of primates and rodents to the exclusion of laurasatheria [41,44].

In our analyses the results seem to favor the basal position of rodents, although the difference with the alternative hypothesis of a clade grouping rodents and primates is not great (Figure 2). From the 14,883 trees in the human phylome with representatives for the three groups (Figure 2), 6,589 (44.3%) show a topology in which rodents are basal, compared to 4,859 (32.6%) and 3,435 (23.1%) trees in which rodents are monophyletic with primates and laurasatheria, respectively. As in the case of arthropods, nematodes and chordates, all possible topologies are fairly represented. Here too, differences in the relative evolutionary rates, and the possible long-branch attraction effect, might have an effect on the high proportion of trees showing rodents at a basal position, since rodent sequences have been shown to have the highest rates of substitutions when compared with primates and artiodactyls [45,46].

### Unikont hypothesis

Among the most difficult problems in the evolution of eukaryotes is resolving the relative branching order of the major eukaryotic groups. The evolutionary distances and the level of sequence divergence involved results in a star-like tree with the major eukaryotic groups branching out in a poorly defined order. Nevertheless, phylogenetic analyses have been used to cluster some of the groups. One such case is the union of amoebozoans and opisthokonts, dubbed the unikonts [47]. Evidence supporting this group comes from phylogenies based on concatenated alignments of up to 149 genes [39] as well as from morphological data. However, this grouping is still not widely accepted among systematicists. In the present analysis a single amoebozoan genome, that of *Dictyostellum discoideum*, has been included, together with representatives from three other major groups, including excavates (*L*. *major*, *P*. *tetraurelia*, *G*. *thetha*), plants (*A*. *thaliana*, *C*. *reinardthii*) and chromoalveolates (*P*. *falciparum*, *P*. *yoelii*). We scanned the phylome for trees supporting the grouping of opisthokonts with each of the other major groups, provided that at least four of the five major groups were represented in the tree (Figure 2). Of the 165 trees in the human phylome including at least four of the five major groups, 64 (39.5%) supported the Unikont hypothesis. The alternative hypotheses of opisthokots being monophyletic with either plants, chromoalveolates or excavates are supported by 58 (35.8%), 31 (19.1%) and 9 (5.6%) trees, respectively. However, differences between the Unikont and the alternative hypotheses are greater when only the 68 trees with high (>0.9) posterior probability in the partition supporting the monophyly are considered. In this case the Unikont hypothesis is consistent, with 42 (61.7%) trees compared to 13, 11 and 2 trees supporting the alternative hypotheses of opisthokonts grouping with plants, chromoalveolates and excavates, respectively.

### Lineage-specific gene duplication

During the course of evolution, gene families can increase their size through events of gene duplication [48]. These events may correspond to massive duplications affecting many genes in the genome at the same time, such as in whole genome duplications (WGDs) or may be restricted to chromosomal segments or specific genes. The idea that gene duplication has played a major role in evolution, acting as a source for novel functions, was originally developed by Ohno [49]. Accumulating evidence now supports this idea. Not only recent genomics surveys have provided evidence for the abundance of duplicated genes in all organisms [50], but it has also been observed that gene duplication is often associated with processes of neo-functionalization and/or sub-functionalization [51].

**Figure 3:**
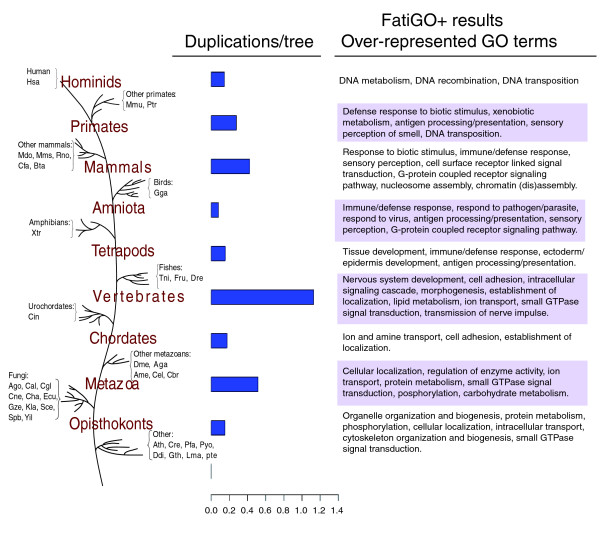
Estimates for the number of duplication events occurred at each major transition in the evolution of the eukaryotes. Species abbreviations are the same as in table 1. Horizontal bars indicate the average number of duplications per gene. Boxes on the right list some of the GO terms of the biological process category that are significantly over-represented compared to the rest of the genome in the set of gene families duplicated at a certain stage. A full list of significantly over represented terms is given as a table in the supplementary material [22].

To quantify the extent of gene duplication that has occurred in the lineages leading to human, we scanned the trees to find duplication events (see Materials and methods) and subsequently mapped them onto a species phylogeny that marks the major branching points in the lineage leading to hominids (Figure 3). The relative number of duplication events per gene at each branching point was estimated by dividing the number of duplication events detected at that stage by the number of trees rooted at a deeper branching point; for example, from a tree rooted on a fungal sequence, only duplications following the split of fungi and metazoans were taken into account.

The highest peak in gene duplication events corresponds to the base of chordate evolution, after the split of urochordates (*Ciona intestinalis*) and vertebrates. This observation is consistent with previous results supporting the existence of at least one round, and probably two rounds, of whole genome duplications before the radiation of vertebrates [52,53], which could explain the increase in phenotypic complexity of vertebrates relative to other chordates such as cephalochordates (amphioxus) and urochordates (*Ciona*). The second largest peak appears at the base of the metazoans, after their split with fungi. The relatively large duplication rate (0.58 duplications per tree) at this point could be interpreted as a result of a WGD at the base of metazoan evolution or, alternatively, an accumulation of smaller scale duplications. To the best of our knowledge, the possibility of a WGD at the base of metazoan evolution has not been proposed in the literature [54] and we believe it deserves some deeper consideration in future analyses. If the WGD scenario is considered, then an extensive gene loss should have followed it, since the duplication rate here is lower than the one found at the base of vertebrate evolution. The alternative scenario would assume a high number of smaller scale duplications that affected more than 50% of the genes. These duplication events would have accumulated over the period of time extending from the split of fungi and metazoans to the split of chordates and other metazoans.

Also remarkable is the relatively high duplication rates found in the lineages leading to mammals, primates and hominids. This suggests that duplications have played a major role in the evolution of these groups, something that has already been noted from comparisons of primate genomes [55].

### Functional trends among duplicated gene sets

The duplication of genes might result in the amplification and/or diversification of the biological processes in which they play a role; if this provides a selective advantage, the duplicated copies will likely be retained. Therefore, inspecting the functions of gene families that have undergone duplication at different evolutionary stages may provide clues about the processes that played roles in the major transitions that occurred during those stages. To detect such functional trends we searched for Gene Ontology (GO) terms that are significantly over-represented in the set of genes that underwent duplications during the different stages of eukaryotic evolution. We performed this analysis automatically with the aid of the program Fatigo+, from the Babelomics suite [56]. At each evolutionary stage (Figure 3) we compared the annotations of the duplicated human genes with those of the rest of the human genome. We selected those terms whose over-representation was significant based on a false discovery rate test (adjusted *p*-value < 0.00001). Due to space limitations we represent only a fraction of the over-represented terms for the category 'biological process' (Figure 3). A complete list of enriched terms in each stage is given in the supplementary material [22]. The present analysis detects over-represented functional categories among genes duplicated at different evolutionary periods. It is, therefore, different from complementary analyses that detect functional shifts and different patterns of amino acid replacement among duplicated pairs [57,58]. Interestingly, these complementary analyses also show differences among functional classes.

In most evolutionary stages, we found several terms from different GO levels and categories that are significantly over-represented. Of these, some are specific to a given evolutionary transition (for example, lipid metabolism in vertebrates), while others are over-represented in a series of consecutive stages (for example, small GTPase signaling cascade). Providing links between the over-represented terms and the functional or morphological transitions characteristic of each stage is not straightforward. Nevertheless, some terms do suggest the expansion of some physiological processes at a given evolutionary time. Terms related to maintenance of complex cellular structures, such as 'organelle organization and biogenesis', 'cytoskeleton', 'cellular organization' or 'cellular localization', are over-represented in genes duplicated before the divergence of fungi and metazoans, suggesting major transitions in cellular organization common to all opisthokonts. The expansion of the process 'small GTPase signal transduction' in almost all major stages from the origin of opisthokonts to the vertebrates indicates a continuous expansion of signaling cascades that is likely related to the increasing level of multi-cellularity and tissue differentiation observed at these evolutionary stages. Similarly, protein families related to 'G-protein coupled receptor signaling pathway' were expanded before the amniota and mammalian radiations. Also remarkable are the consecutive waves of expansion observed for the 'immune response' and related terms. They have occurred at every split from the origin of tetrapods to the origin of primates and suggest an increasing sophistication of the immune system. Xenobiotic metabolism terms are also over-represented in genes duplicated in primates. As noted before [55], the sophistication of the immune response and xenobiotic recognition and detoxification might have facilitated adaptation to changes in food sources and infectious agents.

The specific association of terms such as 'transmission of nerve pulse' or 'nervous system development' with families duplicated just before the vertebrate expansion is consistent with the development of a complex nervous system as compared to that of simpler chordates. Later on, the expansion of 'sensory perception' and related terms in the lineages leading to amniota, mammals and primates indicates increasing sophistication of the senses. Similarly, the term 'epidermis development' is over-represented in genes duplicated in tetrapods. This might be related to major skin modifications, which potentially allowed the conquering of the terrestrial environment by this group.

### Absence of horizontal transfers of eukaryotic genes in the human lineage

The extent and scope of horizontal gene transfer (HGT) events among organisms has been the subject of intense debate [59]. The emerging view is that HGT constitutes an important process of evolution in prokaryotes and that it is more restricted, if not virtually absent, in eukaryotes. However, as more eukaryotic genomes are being sequenced, the number of putative cases of gene transfers in eukaryotes is growing. Reported cases include acquisition of prokaryotic genes [60-62] and transfers of mitochondrial genes between plants [63] and between animals [64]. Horizontal gene transfer in the human genome has been addressed in the past. For instance, after the initial sequencing of the human genome the claim was made that up to 223 bacterial genes, likely acquired by HGT, could be found in the human genome [65]. This claim, however, was later rejected on the basis of phylogenetic analysis [66]. The existence of horizontally transferred genes from other eukaryotes in the human genome has never been reported despite the fact that integrative viral sequences can migrate between vertebrate species and that these viruses can sometimes carry genes within their sequences, making the hypothesis theoretically plausible [67].

The species represented in our phylome include organisms that are tightly linked to human, either because they are pathogens (plasmodium and several fungi), or used as a source of food (cow, yeast). A recent transfer from any of these species to the human genome could, in principle, be detected as a human protein being placed in a 'wrong' phylogenetic context. However, caution must be taken when interpreting phylogenies, since such topologies can also be explained by alternative processes such as multiple gene-loss or lack of phylogenetic resolution.

To find such putative cases we scanned the human phylome to detect trees in which the phylogenetic position of the human seed protein could suggest a possible HGT event. For this purpose we applied a series of increasingly stringent filters. These filters consisted in identifying trees in which: the human seed protein has non-primate proteins as nearest phylogenetic neighbors; such topology cannot be explained simply by the loss of the orthologous sequences in the other primates or multiple losses in mammalian groups; the partition suggesting the HGT is supported by a high posterior probability (>0.9) in the Bayesian analysis; and that partition is also supported by ML analysis. This methodology bears some similarity to that proposed by Hallet *et al*. [68] in that it specifically defines possible scenarios for HGT.

A total of 99 trees (0.47%) passed the first two filters, thus having a topology that could be explained by an HGT event. However, only 8 of these trees had a posterior probability supporting the HGT partition of 0.9 or higher in the Bayesian analysis, and none of these was supported by the ML analyses, indicating that the partitions suggesting the horizontal transfer are not strongly supported.

We interpret these results as a lack of evidence supporting the existence of human genes originating from recent horizontal transfers from the lineages considered and argue that the observed HGT-like topologies are rather the result of phylogenetic artifacts. This interpretation is consistent with the generally adopted view that horizontal gene transfers among multi-cellular eukaryotes is virtually absent due to the existing natural barriers that prevent transferred genes from reaching the germ-line [69].

### Towards a complete catalogue of orthology and paralogy relationships

Although an increasing number of genome-wide experimental datasets is becoming available for human, most experimental analyses are performed in model species such as mouse, fruit fly, yeast and the nematode *Caenorhabditis elegans*. Additionally, for historical or practical reasons, alternative model species are used to investigate specific systems or pathways. Such is the case with the use of *Neurospora crassa*, *Yarrowia lipolytica *and *Bos taurus *models in the characterization of the multiprotein enzyme NADH:Ubiquinone oxidoreductase (Complex I), in which an intricate evolution and the use of different naming schemas in the various species complicate the transfer of knowledge among investigators studying the different model species [70].

Comparative genomics can be used for transferring functional information across species, a process that requires the establishment of evolutionary relationships among genes encoded in the different genomes. Such relationships are best established by means of detecting orthology, rather than just homology. Orthologs are a special case of homologous genes that diverged from a common ancestor through speciation events, in contrast to paralogs, which originate from duplication events [71]. Since orthologs are, relative to paralogs, more likely to share a common function, the correct determination of orthology has deep implications for the transfer of functional information across organisms. This is not, however, the only application of orthology determination. For instance, the establishment of equivalences among genes in different genomes is a pre-requisite for comparing genomics data, something that, in turn, allows the detection of evolutionarily conserved functional associations [72].

The need for detecting orthology at a genome-scale has triggered the development of a variety of automatic approaches that identify orthology relationships by means of similarity searches. The first and still most widely used such method is based on the detection of best reciprocal hits (BRHs), that is, pairs of sequences from different species that are, reciprocally, the best hit of each other in a sequence search [73]. Extensions of the BRH approach include the definition of 'tri-angular' BRH relationships across a minimum of three species [48], and recent implementations thereof, such as Inparanoid [74] or OrthoMCL [75], that include closely related paralogs in the orthologous groups. Although these methods perform reasonably well in most cases, they have been shown to present many drawbacks that can lead to annotation errors or misinterpretation of data [76,77]. More recently, in an attempt to approximate the classic, phylogeny-based approach, several automatic methods have been proposed that delineate orthology relationships from phylogenetic trees. Generally, these methods rely on the detection of duplication and speciation events by comparing the gene tree with the species tree [78]. Several databases have been developed that employ such algorithms to derive orthology relationships from automatically reconstructed trees [79-81]. However, these methods are very sensitive to slight variations in the topology of the gene tree and, when applied at a large-scale, they may perform similarly or even worse than standard pair-wise methods [82]. Some recent developments that use soft-parsimony [83] and model-based approaches [84] for tree-reconciliation allow some level of uncertainty in both the gene-tree and the species-tree.

Considering the high degree of topological diversity observed in the human phylome (see above), we reasoned that any algorithm based on reconciliation with a specific species tree would inevitably infer false duplication events in the trees showing topologies that depart from the canonical species tree. Therefore, we decided to explore an alternative, fully automated approach that does not require a fully resolved species phylogeny and a reconciliation phase. The algorithm (see Materials and methods) uses the level of overlap in the species connected to two related nodes to decide whether their parental node represents a duplication or speciation event. The full list of predicted ortology and paralogy relationships is provided as supplementary material [22].

**Figure 4:**
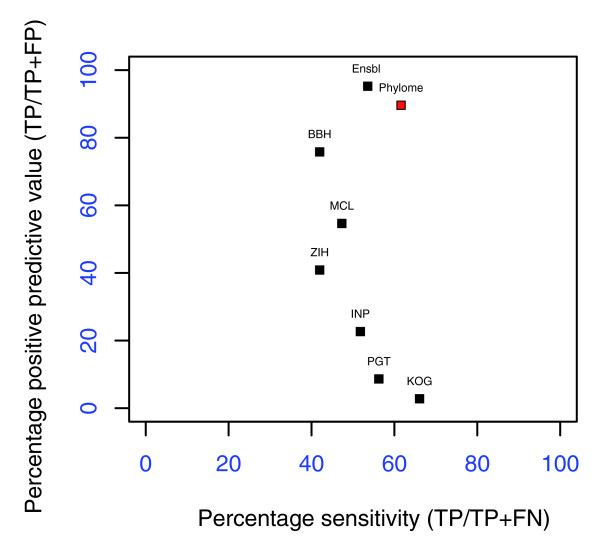
Benchmarking comparison of different orthology inference algorithms. The reference set used in the benchmark of Hulsen *et al*. [82] is taken as a gold standard to compute the number of true positives (TP), false positives (FP) and false negatives (FN) yielded by each method. For each method the sensitivity (S = TP/(TP+FN)) and the positive predictive value (P = TP/(TP + FP)) are computed. Methods described in [82] are indicated as BBH (Best reciprocal hits), MCL (OrthoMCL), ZIH (Z-score 1-hundred.), INP (Inparanoid), PGT (phylogeny-based algorithm used in [95]), KOG (Clusters of eukaryotic orthologous goups). 'Phylome' represents the results of our pipeline and algorithm, and Ensbl the orthology relationships predicted by Ensembl database.

We compared our predictions with those from other algorithms by using a recent reference dataset comprising 67 human-mouse and 45 human-worm orthologous pairs from five multi-gene families [82]. Considering the size of the families and the intricate evolutionary histories involved, this reference set should be considered a highly stringent test. For each of the methods compared we computed the sensitivity, which is a measure of the coverage over the reference set, and the positive predictive value, which is the proportion of correct orthology predictions, that is, the number of true positives over the sum of true positives and false negatives. The results of the benchmark showed narrow differences in terms of sensitivity (Figure 4). All methods are able to predict only about half (40% to 66%) of the orthologous pairs in the reference set. Our method scores second best, with 61.6% sensitivity compared to 66.1% for the clusters of eukaryotic orthologous genes (KOG) method; Ensembl reaches a coverage of 55.57%. As we noted before, this low coverage reflects the inherent difficulty of the reference set, in which manual orthology assignments have taken into account domain organization analysis and other sources of information.

Most remarkable are the big differences encountered in the positive predictive values. These range from 2.8% (KOG) to 86.61% (our algorithm) and 95.24% (Ensembl). Altogether, the results show that phylogeny-based orthology detection methods can provide substantial improvement in terms of positive predictive value when sophisticated phylogenetic pipelines are implemented. Note that the other phylogeny-based method included (Phylogenetic tree, PGT), uses NJ trees. The low rate of false positive prediction achieved by sophisticated phylogeny-based methods makes them especially suited for cases in which orthology prediction is used for the transfer of functional annotations among model species. In such cases, minimizing the level of wrong assignments, which will lead to wrong annotations, is more important than reaching a high coverage at a cost of many false assignments.

## Conclusion

We have shown the feasibility of reconstructing complex phylomes, comprising the evolutionary histories of all genes from a given species and their homologs in dozens of other genomes. The pipeline and genome sampling is fully automated and can easily be tailored for specific needs, therefore paving the way for the reconstructions of other phylomes using different parameters or species sampling. Because of its significance, we have initially applied this pipeline to the human genome. The resulting phylome constitutes a valuable dataset that can be explored by the research community. In the near future we are planning to implement this and other phylomes in a fully searchable database. To illustrate the potential of the human phylome we have performed several analyses, but many others can be envisaged. Overall, our results indicate that there is a great topological diversity affecting the three unresolved scenarios that we have discussed (ecdysozoa versus coelomata, relationships among rodents, primates and laurasatheria and the unikont hypothesis). This and other recent findings [85] reinforce the older view [86] that topological differences among phylogenies of proteins are to be expected even in the absence of HGT and underscore the danger of gene-sampling effects when combining the phylogenetic signals of several genes [34]. We share the view of others [87] that there is an urgent need for improved models of molecular evolution that account for the inherent phylogenetic noise in the protein record and of new genomic characters that are less prone to homoplastic effects. We have found that such noise may eventually produce HGT-like topologies, highlighting the need for stringent cut-offs and alternative tests before an HGT event is assumed.

Mapping speciation and duplication events on the complete phylome has allowed us to derive a comprehensive set of orthology and paralogy relationships among the genomes involved. The results obtained in the benchmark analysis show that although automatic methods for phylogeny-based detection of orthology are progressing in the right direction, there is still room for improvement both in the algorithms and the quality of the trees. Taking into account the levels of topological diversity mentioned above, it follows that the algorithms for phylogeny-based orthology detection need to cope with levels of topological uncertainty.

The results obtained in the present analysis are consistent with the existence of at least one round of whole genome duplication occurring before the radiation of verebrates [49]. If a relatively high level of gene loss in eukaryotic genomes is assumed [88], the finding of an average level of duplication events per tree higher than one would indeed favor the scenario of two rounds of whole genome duplication at that evolutionary stage (2R hypothesis).

## Materials and methods

### Sequence data

Proteomes derived from 39 fully sequenced eukaryotic genomes (Table 1) were downloaded from Ensembl v36 [12] and the Integr8 database at EBI [89], except those of *Candida albicans *[90]*N. crassa *[91] and *C. reinhardtii *[92]. Wherever mitochondrial proteins were not included in the gene set per species, these were downloaded separately from the NCBI eukaryotic organelles site. Mitochondrial genomes from *Caenorhabditis briggsae*, *Giberella zeae*, *Debaromyces hansenii *and *Leishmania major *have apparently not been deposited in the public databases and, therefore, are missing from this study. The final proteome database contains 542,423 unique protein sequences from 39 different genomes (Table 1).

### Database searches

For each human protein a Smith-Waterman [93] search was performed against the above-mentioned proteome database to retrieve a set of proteins with a significant similarity (E-value < 10^-3^). Only sequences that aligned with a continuous region longer than 50% of the query sequence were selected.

### Multiple sequence alignment and phylogenetic reconstructions

Sets of homologous protein sequences were aligned using MUSCLE 3.6 [94]. Positions in the alignment with gaps in more than 10% of the sequences were eliminated before the phylogenetic analysis, unless this procedure removed more than one-third of the positions in the alignment. In such cases the percentage of sequences with gaps allowed was automatically increased until at least two-thirds of the initial positions were conserved.

NJ trees were derived using scoredist distances as implemented in BioNJ [18]. ML trees were derived from the alignments using PhyML v2.4.4 [19]. For each protein family ML trees were reconstructed with four different evolutionary models (JTT, WAG, BLOSUM62 and VT), except for the 13 mitochondrially encoded proteins in which the mtREV model was also used. In all cases a discrete gamma-distribution model with four rate categories plus invariant positions was used, the gamma parameter and the fraction of invariant positions were estimated from the data. The evolutionary model best fitting the data was determined by comparing the likelihood of the used models according to the AIC criterion [20].

To obtain support values of all tree partitions, the ML tree produced by the best-fitting model was used as a seed for a Bayesian analysis by running Mr Bayes [21] for 100,000 generations in two runs of two chains each, using the best-fitting model, as determined in the ML analysis, but allowing branch swapping and re-estimation of the gamma distribution parameters. The posterior probability of each tree partition was estimated by sampling the trees every 100 generations after discarding the first 25%. This approach to obtain support values was faster than performing standard bootstrap analysis with PhyML. Starting the MrBayes runs with an already optimized tree resulted in fair levels of convergence being reached after fewer generations than in standard MrBayes analyses.

A final tree produced by this Bayesian reconstruction consists of a consensus phylogeny, using the 'halfcompat' option in MrBayes, in which partitions with a posterior probability lower than 0.5 are collapsed. The alignments and all trees produced for each human protein are made available as supplemental information [22]. Unless stated otherwise the consensus tree produced by MrBayes analysis was used in all analyses.

### Inference of duplication and speciation events and orthology assignment using a novel algorithm independent of species-tree reconciliation

We used a phylogeny-based algorithm to detect duplication and speciation events on the trees. In contrast to alternative phylogeny-based methods that use reconciliation of the gene tree with the species tree to infer duplication events, our approach does not require any previous fully resolved species topology. The only evolutionary information required is that used to root the trees to define a polarity so each internal node is connected to two children nodes. The orthology prediction algorithm was run independently for each human gene using the tree generated using its protein sequence as a seed. The algorithm was implemented in a series of python scripts specifically developed for this project.

To map duplication and speciation events on an internal node of the tree, the algorithm proceeds as follows. First, two tree partitions are defined that contain the sequences connected to each of the two children nodes. Second, a species-overlap score is defined between the two partitions as follows: species common to both partitions/species in any of the partitions. Third, if the score is higher than a given threshold the node is mapped as a duplication event, otherwise it is considered a speciation event. In the present study the species-overlap threshold was set to 0.0 - that is, no common species between the two partitions were allowed - because this produced the best results in the benchmark. The algorithm does so for all internal nodes in the tree. Once all the nodes in the tree are marked as a duplication or speciation event, the algorithm establishes orthology relationships between the seed protein and other proteins in the tree. For each protein, the algorithm tracks the nodes that connect it to the seed protein and establishes an orthology relationship only if this connection proceeds exclusively through speciation nodes, disregarding intra-specific duplications. After mapping speciation and duplication nodes onto the phylogeny, several situations may arise in which orthology relationships are not one-to-one relationships, but rather one-to-many or many-to-many.

To root the trees the following procedure was used. The species present in the tree were grouped according to the branching pattern of the tree in Figure 3; thus, non-opisthokont species constitute the deepest group, followed by 'fungi', 'other metazoans', 'urochordates', and so on. Among the sequences belonging to the deepest group with representatives in the tree, the one with the longest distance to the seed protein was chosen as the out-group.

### Assigning duplications to different evolutionary periods

The duplication events detected by the algorithm described above can be assigned to different evolutionary periods by examining the species represented after the duplication event. To do so we used as a reference a set of clearly defined phylogenetic relationships that mark the major branching points in the lineage leading to hominids (Figure 3). For each duplication event, all the species represented after the duplication node are tracked and the duplication is assigned to the deepest branching point in the reference tree that contains all these species. For instance, if only sequences from mammals and fishes are found after the duplication event, this duplication is assigned to the branching point that is at the base of vertebrates.

The orthology detection algorithm can detect only duplications that occurred after the root of the tree; for example, if a tree is rooted in a fungal sequence, only duplications that occurred in the metazoan lineage could be detected. Therefore, to compare the results obtained at the different evolutionary stages we computed the relative number of duplication events per gene at each branching point. This was done by dividing the number of duplication events mapped at a particular evolutionary stage by the number of trees rooted at a deeper branching point; for example, duplications that occurred at the base of metazoans were divided by the number of trees rooted on either a fungal or a non-opisthokont sequence.

### Topology scanning algorithm

The algorithm used here to search for specific topologies within the phylome is described elsewhere [9]. In brief, from an un-rooted tree the algorithm generates all possible partitions that contain the seed sequence. That is, the algorithm proceeds sequentially throughout all internal edges of the tree. At each internal edge it generates two partitions, of which only one contains the seed sequence. The species represented in each such partition are tracked and those trees with a partition fulfilling a set of rules defined by the user are selected. The set of rules defined by the user are defined as a set of species that are allowed in a partition, and rules can be combined so that specific evolutionary scenarios are defined. For instance, a partition supporting the grouping of rodents and primates to the exclusion of laurasatherians can be defined as a partition containing any sequence (s) from primates (*Homo sapiens*, *Macacca mulata*, *Pan troglodites*) and any sequence (s) from rodents (*Mus musculus*, *Rattus norvergicus*) within a larger partition that contains these sequence plus any sequence (s) from Laurasatherians (*Canis familiaris*, *Bos taurus*). Sequences from other species are not allowed in the partition and the presence of the seed sequence in the partition is required. This algorithm has been implemented in a series of Python scripts developed for this project.

In the topology scanning analyses presented here we discarded the trees based on alignments in which less than 100 columns were left after applying the gap filter. This procedure eliminated 1,714 (7.9%) from the total phylome.

### Benchmarking

The reference set used in a recent benchmark of orthology assignment methods [82] is used to compute the number of true positives (TPs), false positives (FPs) and false negatives (FNs) yielded by each method. For each method the sensitivity, S = TP/(TP + FN), and the positive predictive value, P = TP/(TP + FP), were computed.

## Additional data files

The following additional data are available with the on-line version of this paper. Additional data file 1 is a table listing the over-represented GO terms in the duplicatons depicted in Figure 3. Additional data file 2 is a table listing the orthologs predicted for every human protein.

## Supplementary Material

Additional data file 1 Over-represented GO terms in the duplicatons depicted in Figure 3 Click here for file

Additional data file 2 Orthologs predicted for every human protein Click here for file

## References

[B1] McPherson JD, Marra M, Hillier L, Waterston RH, Chinwalla A, Wallis J, Sekhon M, Wylie K, Mardis ER, Wilson RK (2001). A physical map of the human genome.. Nature.

[B2] Venter JC, Adams MD, Myers EW, Li PW, Mural RJ, Sutton GG, Smith HO, Yandell M, Evans CA, Holt RA (2001). The sequence of the human genome.. Science.

[B3] Suzuki Y, Sugano S (2006). Transcriptome analyses of human genes and applications for proteome analyses.. Curr Protein Pept Sci.

[B4] Humphery-Smith I (2004). A human proteome project with a beginning and an end.. Proteomics.

[B5] Gandhi TK, Zhong J, Mathivanan S, Karthick L, Chandrika KN, Mohan SS, Sharma S, Pinkert S, Nagaraju S, Periaswamy B (2006). Analysis of the human protein interactome and comparison with yeast, worm and fly interaction datasets.. Nat Genet.

[B6] Nielsen J, Oliver S (2005). The next wave in metabolome analysis.. Trends Biotechnol.

[B7] Benner SA (2003). Interpretive proteomics - finding biological meaning in genome and proteome databases.. Adv Enzyme Regul.

[B8] Sicheritz-Ponten T, Andersson SG (2001). A phylogenomic approach to microbial evolution.. Nucleic Acids Res.

[B9] Gabaldón T, Huynen MA (2003). Reconstruction of the proto-mitochondrial metabolism.. Science.

[B10] Gabaldón T (2005). Evolution of proteins and proteomes, a phylogenetics approach.. Evolutionary Bioinformatics Online.

[B11] Huynen MA, Gabaldon T, Snel B (2005). Variation and evolution of biomolecular systems: searching for functional relevance.. FEBS Lett.

[B12] Birney E, Andrews D, Caccamo M, Chen Y, Clarke L, Coates G, Cox T, Cunningham F, Curwen V, Cutts T (2006). Ensembl 2006.. Nucleic Acids Res.

[B13] Li H, Coghlan A, Ruan J, Coin LJ, Heriche JK, Osmotherly L, Li R, Liu T, Zhang Z, Bolund L (2006). TreeFam: a curated database of phylogenetic trees of animal gene families.. Nucleic Acids Res.

[B14] Duret L, Mouchiroud D, Gouy M (1994). HOVERGEN: a database of homologous vertebrate genes.. Nucleic Acids Res.

[B15] Roth C, Betts MJ, Steffansson P, Saelensminde G, Liberles DA (2005). The Adaptive Evolution Database (TAED): a phylogeny based tool for comparative genomics.. Nucleic Acids Res.

[B16] Blackstone NW, Green DR (1999). The evolution of a mechanism of cell suicide.. Bioessays.

[B17] Fisher SE, Marcus GF (2006). The eloquent ape: genes, brains and the evolution of language.. Nat Rev Genet.

[B18] Gascuel O (1997). BIONJ: an improved version of the NJ algorithm based on a simple model of sequence data.. Mol Biol Evol.

[B19] Guindon S, Gascuel O (2003). A simple, fast, and accurate algorithm to estimate large phylogenies by maximum likelihood.. Syst Biol.

[B20] Akaike H, Institute of Electrical & Electronics Engineers (1973). Information theory and extension of the maximum likelihood principle.. Proceedings of the 2nd International Symposium on Information Theory: 1973; Budapest, Hungary.

[B21] Ronquist F, Huelsenbeck JP (2003). MrBayes 3: Bayesian phylogenetic inference under mixed models.. Bioinformatics.

[B22] Supplementary material. http://bioinfo.cipf.es/data/human_phylome/human_phylome.html.

[B23] Keane TM, Creevey CJ, Pentony MM, Naughton TJ, McLnerney JO (2006). Assessment of methods for amino acid matrix selection and their use on empirical data shows that *ad hoc *assumptions for choice of matrix are not justified.. BMC Evol Biol.

[B24] Bruno WJ, Halpern AL (1999). Topological bias and inconsistency of maximum likelihood using wrong models.. Mol Biol Evol.

[B25] Buckley TR, Cunningham CW (2002). The effects of nucleotide substitution model assumptions on estimates of nonparametric bootstrap support.. Mol Biol Evol.

[B26] Jones DT, Taylor WR, Thornton JM (1992). The rapid generation of mutation data matrices from protein sequences.. Comput Appl Biosci.

[B27] Henikoff S, Henikoff JG (1992). Amino acid substitution matrices from protein blocks.. Proc Natl Acad Sci USA.

[B28] Whelan S, Goldman N (2001). A general empirical model of protein evolution derived from multiple protein families using a maximum-likelihood approach.. Mol Biol Evol.

[B29] Müller T, Vingron M (2000). Modeling amino acid replacement.. J Comput Biol.

[B30] Adachi J, Hasegawa M (1996). Model of amino acid substitution in proteins encoded by mitochondrial DNA.. J Mol Evol.

[B31] Keeling PJ, Burger G, Durnford DG, Lang BF, Lee RW, Pearlman RE, Roger AJ, Gray MW (2005). The tree of eukaryotes.. Trends Ecol Evol.

[B32] Delsuc F, Brinkmann H, Philippe H (2005). Phylogenomics and the reconstruction of the tree of life.. Nat Rev Genet.

[B33] Jeffroy O, Brinkmann H, Delsuc F, Philippe H (2006). Phylogenomics: the beginning of incongruence?. Trends Genet.

[B34] Dagan T, Martin W (2006). The tree of one percent.. Genome Biol.

[B35] Aguinaldo AM, Turbeville JM, Linford LS, Rivera MC, Garey JR, Raff RA, Lake JA (1997). Evidence for a clade of nematodes, arthropods and other moulting animals.. Nature.

[B36] Dopazo H, Dopazo J (2005). Genome-scale evidence of the nematode-arthropod clade.. Genome Biol.

[B37] Ciccarelli FD, Doerks T, von Mering C, Creevey CJ, Snel B, Bork P (2006). Toward automatic reconstruction of a highly resolved tree of life.. Science.

[B38] Wolf YI, Rogozin IB, Koonin EV (2004). Coelomata and not Ecdysozoa: evidence from genome-wide phylogenetic analysis.. Genome Res.

[B39] Philippe H, Snell EA, Bapteste E, Lopez P, Holland PW, Casane D (2004). Phylogenomics of eukaryotes: impact of missing data on large alignments.. Mol Biol Evol.

[B40] Blair JE, Hedges SB (2005). Molecular phylogeny and divergence times of deuterostome animals.. Mol Biol Evol.

[B41] Murphy WJ, Pevzner PA, O'Brien SJ (2004). Mammalian phylogenomics comes of age.. Trends Genet.

[B42] Kullberg M, Nilsson MA, Arnason U, Harley EH, Janke A (2006). Housekeeping genes for phylogenetic analysis of eutherian relationships.. Mol Biol Evol.

[B43] Misawa K, Janke A (2003). Revisiting the Glires concept - phylogenetic analysis of nuclear sequences.. Mol Phylogenet Evol.

[B44] Thomas JW, Touchman JW, Blakesley RW, Bouffard GG, Beckstrom-Sternberg SM, Margulies EH, Blanchette M, Siepel AC, Thomas PJ, McDowell JC (2003). Comparative analyses of multi-species sequences from targeted genomic regions.. Nature.

[B45] Ohta T (1995). Synonymous and nonsynonymous substitutions in mammalian genes and the nearly neutral theory.. J Mol Evol.

[B46] Zhang J (2000). Rates of conservative and radical nonsynonymous nucleotide substitutions in mammalian nuclear genes.. J Mol Evol.

[B47] Cavalier-Smith T (2002). The phagotrophic origin of eukaryotes and phylogenetic classification of Protozoa.. Int J Syst Evol Microbiol.

[B48] Tatusov RL, Koonin EV, Lipman DJ (1997). A genomic perspective on protein families.. Science.

[B49] Ohno S (1970). Evolution by Gene Duplication.

[B50] Vogel C, Chothia C (2006). Protein family expansions and biological complexity.. PLoS Comput Biol.

[B51] Roth C, Rastogi S, Arvestad L, Dittmar K, Light S, Ekman D, Liberles DA (2007). Evolution after gene duplication: models, mechanisms, sequences, systems, and organisms.. J Exp Zoolog B Mol Dev Evol.

[B52] Panopoulou G, Hennig S, Groth D, Krause A, Poustka AJ, Herwig R, Vingron M, Lehrach H (2003). New evidence for genome-wide duplications at the origin of vertebrates using an amphioxus gene set and completed animal genomes.. Genome Res.

[B53] Blomme T, Vandepoele K, De Bodt S, Simillion C, Maere S, Van de Peer Y (2006). The gain and loss of genes during 600 million years of vertebrate evolution.. Genome Biol.

[B54] Meyer A (2003). Molecular evolution: Duplication, duplication.. Nature.

[B55] Bailey JA, Eichler EE (2006). Primate segmental duplications: crucibles of evolution, diversity and disease.. Nat Rev Genet.

[B56] Al-Shahrour F, Minguez P, Tarraga J, Montaner D, Alloza E, Vaquerizas JM, Conde L, Blaschke C, Vera J, Dopazo J (2006). BABELOMICS: a systems biology perspective in the functional annotation of genome-scale experiments.. Nucleic Acids Res.

[B57] Abhiman S, Sonnhammer EL (2005). FunShift: a database of function shift analysis on protein subfamilies.. Nucleic Acids Res.

[B58] Seoighe C, Johnston CR, Shields DC (2003). Significantly different patterns of amino acid replacement after gene duplication as compared to after speciation.. Mol Biol Evol.

[B59] Kurland CG, Canback B, Berg OG (2003). Horizontal gene transfer: a critical view.. Proc Natl Acad Sci USA.

[B60] Andersson JO, Sjogren AM, Davis LA, Embley TM, Roger AJ (2003). Phylogenetic analyses of diplomonad genes reveal frequent lateral gene transfers affecting eukaryotes.. Curr Biol.

[B61] Ricard G, McEwan NR, Dutilh BE, Jouany JP, Macheboeuf D, Mitsumori M, McIntosh FM, Michalowski T, Nagamine T, Nelson N (2006). Horizontal gene transfer from Bacteria to rumen Ciliates indicates adaptation to their anaerobic, carbohydrates-rich environment.. BMC Genomics.

[B62] Goldsmith MR, Shimada T, Abe H (2005). The genetics and genomics of the silkworm, Bombyx mori.. Annu Rev Entomol.

[B63] Bergthorsson U, Adams KL, Thomason B, Palmer JD (2003). Widespread horizontal transfer of mitochondrial genes in flowering plants.. Nature.

[B64] Alvarez N, Benrey B, Hossaert-McKey M, Grill A, McKey D, Galtier N (2006). Phylogeographic support for horizontal gene transfer involving sympatric bruchid species.. Biol Direct.

[B65] Lander ES, Linton LM, Birren B, Nusbaum C, Zody MC, Baldwin J, Devon K, Dewar K, Doyle M, FitzHugh W (2001). Initial sequencing and analysis of the human genome.. Nature.

[B66] Salzberg SL, White O, Peterson J, Eisen JA (2001). Microbial genes in the human genome: lateral transfer or gene loss?. Science.

[B67] Bromham L (2002). The human zoo: endogenous retroviruses in the human genome.. Trends Ecol Evol.

[B68] Hallet M, Lagergren J, Tofigh A (2004). Simultaneous identification of duplications and lateral transfers.. Proceedings of the Eighth Annual International Conference on Research In Computational Molecular Biology: 2004; San Diego, California, USA.

[B69] Kurland CG (2005). What tangled web: barriers to rampant horizontal gene transfer.. Bioessays.

[B70] Gabaldón T, Rainey D, Huynen MA (2005). Tracing the evolution of a large protein complex in the eukaryotes, NADH:ubiquinone oxidoreductase (Complex I).. J Mol Biol.

[B71] Fitch WM (1970). Distinguishing homologous from analogous proteins.. Syst Zool.

[B72] Gabaldón T, Huynen MA (2004). Prediction of protein function and pathways in the genome era.. Cell Mol Life Sci.

[B73] Huynen MA, Bork P (1998). Measuring genome evolution.. Proc Natl Acad Sci USA.

[B74] O'Brien KP, Remm M, Sonnhammer EL (2005). Inparanoid: a comprehensive database of eukaryotic orthologs.. Nucleic Acids Res.

[B75] Li L, Stoeckert CJ, Roos DS (2003). OrthoMCL: identification of ortholog groups for eukaryotic genomes.. Genome Res.

[B76] Eisen JA (1998). Phylogenomics: improving functional predictions for uncharacterized genes by evolutionary analysis.. Genome Res.

[B77] Koonin EV (2005). Orthologs, paralogs, and evolutionary genomics.. Annu Rev Genet.

[B78] Zmasek CM, Eddy SR (2001). A simple algorithm to infer gene duplication and speciation events on a gene tree.. Bioinformatics.

[B79] Zmasek CM, Eddy SR (2002). RIO: analyzing proteomes by automated phylogenomics using resampled inference of orthologs.. BMC Bioinformatics.

[B80] Dehal PS, Boore JL (2006). A phylogenomic gene cluster resource: the Phylogenetically Inferred Groups (PhIGs) database.. BMC Bioinformatics.

[B81] Chiu JC, Lee EK, Egan MG, Sarkar IN, Coruzzi GM, DeSalle R (2006). OrthologID: automation of genome-scale ortholog identification within a parsimony framework.. Bioinformatics.

[B82] Hulsen T, Huynen MA, de Vlieg J, Groenen PM (2006). Benchmarking ortholog identification methods using functional genomics data.. Genome Biol.

[B83] Berglund-Sonnhammer AC, Steffansson P, Betts MJ, Liberles DA (2006). Optimal gene trees from sequences and species trees using a soft interpretation of parsimony.. J Mol Evol.

[B84] Arvestad L, Berglund AC, Lagergren J, Sennblad B (2003). Bayesian gene/species tree reconciliation and orthology analysis using MCMC.. Bioinformatics.

[B85] Rokas A, Williams BL, King N, Carroll SB (2003). Genome-scale approaches to resolving incongruence in molecular phylogenies.. Nature.

[B86] Penny D, Foulds LR, Hendy MD (1982). Testing the theory of evolution by comparing phylogenetic trees constructed from five different protein sequences.. Nature.

[B87] Rokas A, Carroll SB (2006). Bushes in the tree of life.. PLoS Biol.

[B88] Gabaldón T, Huynen MA (2005). Lineage-specific gene loss following mitochondrial endosymbiosis and its potential for function prediction in eukaryotes.. Bioinformatics.

[B89] Pruess M, Kersey P, Apweiler R (2005). The Integr8 project - a resource for genomic and proteomic data.. In Silico Biol.

[B90] Candida Genome Database. http://www.candidagenome.org.

[B91] *Neurospora crassa *at MIT. http://www.broad.mit.edu/annotation/fungi/neurospora.

[B92] Chlamydomonas genome at JGI. http://genome.jgi-psf.org/chlamy.

[B93] Smith TF, Waterman MS (1981). Identification of common molecular subsequences.. J Mol Biol.

[B94] Edgar RC (2004). MUSCLE: a multiple sequence alignment method with reduced time and space complexity.. BMC Bioinformatics.

[B95] van Noort V, Snel B, Huynen MA (2003). Predicting gene function by conserved co-expression.. Trends Genet.

